# P-1213. Activity of Tebipenem Against Enterobacterales, Including Molecularly Characterized Clinical Isolates Causing Urinary Tract and Bloodstream Infections from the United States in 2023

**DOI:** 10.1093/ofid/ofaf695.1406

**Published:** 2026-01-11

**Authors:** Renuka Kapoor, Timothy Doyle, Zachary Kockler, Rodrigo mendes, Mariana Castanheira, Didem Torumkuney, Ian A Critchley

**Affiliations:** GSK, Atlanta, Georgia; Element Materials Technology/Jones Microbiology Institute, North Liberty, IA; Element Iowa City (JMI Laboratories), North Liberty, Iowa; Element Iowa City, North Liberty, Iowa; Element, North Liberty, IA; GSK, Atlanta, Georgia; Spero Therapeutics, Cambridge, Massachusetts

## Abstract

**Background:**

Tebipenem pivoxil hydrobromide (TBP) (formerly SPR994) is in clinical development as the potential first oral broad-spectrum carbapenem agent in the US for the treatment of complicated urinary tract infections (cUTI) and acute pyelonephritis (AP). This study reports on the *in vitro* activity of TBP and comparator agents against molecularly characterized Enterobacterales isolates recovered from UTI and bloodstream infections (BSI) in the US, including ESBL and carbapenemase (CP) producing isolates.

**Figure:**
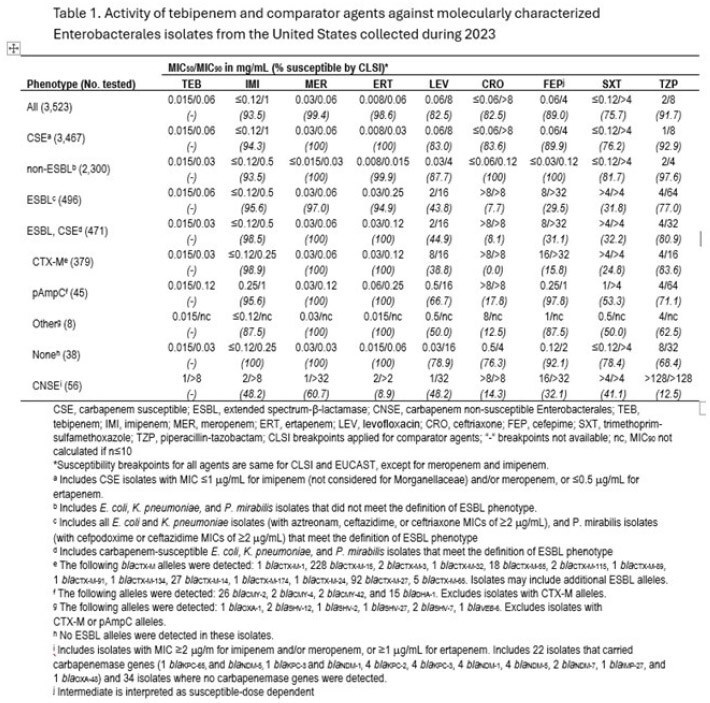

**Methods:**

A total of 3,523 Enterobacterales isolates collected from the US in 2023 were included. (UTI, 74.2% (2,614), BSI, 25.8% (909)). Isolates were tested for susceptibility (S) by CLSI reference broth microdilution method. *E. coli* and *K. pneumoniae* with aztreonam (ATM), ceftazidime (CAZ), or ceftriaxone (CRO) MICs of ≥2 µg/mL, and *P. mirabilis* with cefpodoxime (CPD) or CAZ MICs of ≥2 µg/mL were classified as ESBL phenotype. Isolates with MIC ≥2 µg/mL for imipenem (IMI) and/or meropenem (MER), or ≥1 µg/mL for ertapenem (ERT), were categorized as carbapenem-nonsusceptible (CNSE) phenotype. Isolates that met these criteria were screened for plasmid-mediated AmpC (pAmpC), ESBL, and CP genes.

**Results:**

A total of 14.1% (496/3,523) of isolates were identified with an ESBL phenotype and 13.3% (471/3,523) were ESBL, CSE phenotype (Table). Of the latter, 91.7% (432/471) carried ESBL and/or pAmpC genes. TBP had MIC_50/90_ of 0.015/0.03 µg/mL against this subset, with those for IMI (MIC_50/90_, ≤0.12/0.5 µg/mL), MER (MIC_50/90_, 0.03/0.06 µg/mL) and ERT (MIC_50/90_, 0.03/0.12 µg/mL). The S to other comparators was below 81%. The CNSE phenotype accounted for only 1.6% (56/3,523) of isolates, and 39.3% (22/56) carried CP. TBP displayed MIC_50/90_ of 1/ >8 µg/mL. IMI (MIC_50/90_, 2/ >8 µg/mL), MER (MIC_50/90_, 1/ >32 µg/mL) and ERT (MIC_50/90_, 2/ >2 µg/mL) were active against 48%, 61% and 9% of the CNSE subset, respectively, while the S of oral comparators was ≤48%.

**Conclusion:**

TBP displayed MICs similar to those for overall isolates against ESBL-producing Enterobacterales isolates from UTIs and BSIs in US medical centers. These results indicate that TBP has activity comparable to IV carbapenems and has the potential for use as oral treatment option for cUTI and AP.

**Disclosures:**

Renuka Kapoor, PhD, GSK: Employee|GSK: Stocks/Bonds (Public Company) Mariana Castanheira, PhD, Melinta Therapeutics: Advisor/Consultant|Melinta Therapeutics: Grant/Research Support Didem Torumkuney, PhD, GSK: Stocks/Bonds (Public Company) Ian A. Critchley, PhD, Spero Therapeutics: Stocks/Bonds (Public Company)

